# Whole Genome Sequence Analysis Reveals Lower Diversity and Frequency of Acquired Antimicrobial Resistance (AMR) Genes in *E. coli* From Dairy Herds Compared With Human Isolates From the Same Region of Central Zambia

**DOI:** 10.3389/fmicb.2019.01114

**Published:** 2019-05-31

**Authors:** Geoffrey Mainda, Nadejda Lupolova, Linda Sikakwa, Emily Richardson, Paul R. Bessell, Sydney K. Malama, Geoffrey Kwenda, Mark P. Stevens, Barend M. deC. Bronsvoort, John B. Muma, David L. Gally

**Affiliations:** ^1^Royal (Dick) School of Veterinary Studies, University of Edinburgh, Edinburgh, United Kingdom; ^2^Department of Veterinary Services, Ministry of Fisheries and Livestock, Lusaka, Zambia; ^3^Department of Disease Control, School of Veterinary Medicine, University of Zambia, Lusaka, Zambia; ^4^MicrobesNG, University of Birmingham, Birmingham, United Kingdom

**Keywords:** AMR, *E. coli*, antibiotic, cattle, dairy

## Abstract

Antibiotic treatment of sick dairy cattle is critical for the sustainability of this production system which is vital for food security and societal prosperity in many low and middle-income countries. Given the increasingly high levels of antibiotic resistance worldwide and the challenge this presents for the treatment of bacterial infections, the rational use of antibiotics in humans and animals has been emphatically recommended in the spirit of a “One Health” approach. The aim of this study was to characterize antimicrobial resistance (AMR) genes and their frequencies from whole genome sequences of *Escherichia coli* isolated from both dairy cattle and human patients in central Zambia. Whole genome sequences of *E. coli* isolates from dairy cattle (*n* = 224) and from patients at a local hospital (*n* = 73) were compared for the presence of acquired AMR genes. In addition we analyzed the publicly available genomes of 317 human *E. coli* isolates from over the wider African continent. Both acquired antibiotic resistance genes and phylogroups were identified from *de novo* assemblies and SNP based phylogenetic analyses were used to visualize the distribution of resistance genes in *E. coli* isolates from the two hosts. Greater acquired AMR gene diversity was detected in human compared to bovine *E. coli* isolates across multiple classes of antibiotics with particular resistance genes for extended-spectrum beta lactamases (ESBL), quinolones, macrolides and fosfomycin only detected in *E. coli* genomes of human origin. The striking difference was that the Zambian or wider African human isolates were significantly more likely to possess multiple acquired AMR genes compared to the Zambian dairy cattle isolates. The median number of resistance genes in the Zambian cattle cohort was 0 (0–1 interquartile range), while in the Zambian human and wider African cohorts the medians and interquartile ranges were 6 (4–9) and 6 (0–8), respectively. The lower frequency and reduced diversity of acquired AMR genes in the dairy cattle isolates is concordant with relatively limited antibiotic use that we have documented in this region, especially among smallholder farmers. The relatively distinct resistant profiles in the two host populations also indicates limited sharing of strains or genes.

## Introduction

Antimicrobial resistance (AMR), in particular to antibiotics, has placed a huge burden on public health delivery systems. AMR contributes to treatment failure or prolonged hospitalization of human patients and the global extent of the issue has been captured by WHO reports including a definition of the priority resistant pathogens (Tacconelli et al., 2013; World Health Organization [WHO], 2017a,b). These include multi-drug resistant Enterobacteriaceae, including *Escherichia coli*, which combined with its relative ease of isolation and capacity for gene exchange, makes it an important sentinel organism to understand the epidemiology of AMR in different environments and animal hosts. AMR is an increasing threat to human and animal life and this is exacerbated in regions of the globe by inadequate health facility infrastructures and lower sanitation and hygiene.

Antibiotic accessibility is variable across the planet, for both economic and legislative reasons. The emergence and spread of AMR in animals, humans and the environment is extremely complex and it is very difficult to demonstrate where reduced use would be most effective in terms of restricting AMR (Woolhouse et al., 2015). As a consequence, a precautionary principle is being applied in livestock (O’Neill, 2014), including legislation, rotational practices, and promotion of the use of disease preventive measures such as vaccines to reduce the use of antibiotics, especially those of last resort in the treatment of human infections. While this must be supported, antibiotics are critical for the effective treatment of livestock diseases and help preserve the economic viability of small-scale livestock practices in many low and middle income countries. As such there is an argument that “the genie is out of the bottle” and the sporadic use of 1st and 2nd generation antimicrobials for production animals to treat infections is critical and is unlikely to impact on human infection treatments (van Bunnik and Woolhouse, 2017). Toward this, more information is required about resistance genes in livestock species and humans in different settings across the globe.

In Zambia, farming at different scales is central to the country’s economy and the livelihoods of many smallholders and their families and communities. There is limited information from Zambia on AMR levels in farmed livestock. In one recent study (Chishimba et al., 2016) just over 20% of *E. coli* isolates from chickens surveyed were shown to contain extended-spectrum β-lactamases (ESBLs) indicating they are a reservoir of important AMR genes, although the risk of strain and/or gene transfer to humans is unknown. Our recent work in central Zambia established that antibiotic use in the dairy sector was relatively well controlled and phenotypic AMR in bovine *E. coli* was statistically associated with prescribing trends, use of introduced exotic (higher production) breeds, and treatment of lumpy skin disease but not bacterial mastitis (Mainda et al., 2015). Recent developments in sequencing have revolutionized the diagnosis of infectious and non-infectious diseases in public health and, when applied in the context of AMR, makes it possible to identify different resistance genes and also to generate refined dissemination genetic maps and study the phylogenetics of the resistomes of the microorganisms involved (Crofts et al., 2017; van Bunnik and Woolhouse, 2017; Baker et al., 2018). Here, the sequencing of *E. coli* isolates from dairy cattle from over 100 smallholdings/farms within a radius of 120 km around Lusaka provided an opportunity to analyze the AMR genotypes of the isolates. During this work, we were informed of a small-scale study collecting *E. coli* from human patients presenting with diarrhea at a referral hospital in Lusaka (University Teaching Hospital). This allowed a comparison of the frequency and diversity of acquired AMR genes associated with *E. coli* in the two hosts in this region. This analysis was then extended to include *E. coli* genomes from humans available from the Enterobase (Alikhan et al., 2018) database across from multiple African countries.

## Materials and Methods

### Sample Selection and Whole Genome Sequencing

The cattle isolates were from a previously described study, with the main sampling from 376 dairy cattle covering 104 farms in Lusaka and surrounding areas over a 4-month period in early 2014 (Mainda et al., 2015). A total of 371 *E. coli* isolates were tested phenotypically for resistance to a panel of six different antibiotics and 61/371 (16.4%) were positive for resistance to at least one of the antibiotic classes. The resistance prevalence estimates were as published previously (Mainda et al., 2015) and the proportions of isolates resistant or susceptible to the tested antibiotics are shown as Supplementary Data Sheet S1. All 61 isolates exhibiting phenotypic resistance were sequenced to capture their resistance gene diversity. For comparison, a further 125 randomly sampled Zambian cattle *E. coli* isolates with no phenotypic resistance were also sequenced from the remaining 310 isolates. A previously published study (Mainda et al., 2016) examining the zoonotic threat of Shiga toxin positive *E. coli* from these Zambian cattle samples involved the sequencing of 41 of these isolates (without phenotypic resistance) and so these genomes were also included in our analysis. Overall there were 227/371 cattle isolates that were genome sequenced and 224 were of sufficient quality for analysis of acquired AMR genes. This breakdown of *E. coli* strains for sequencing is shown as Supplementary Data Sheet S2. We appreciate that the *E. coli* isolates selected for sequencing from cattle overestimates the actual levels of phenotypic resistance (32.2% compared to 16.4%) but ensured that the bovine *E. coli* AMR gene diversity was captured and there was phylogenetic context for both resistant and sensitive bovine *E. coli* isolates.

*Escherichia coli* were also isolated from patients with diarrhea at the University Teaching Hospital in Lusaka using the methodologies applied to our cattle work (Mainda et al., 2015). Lusaka is the central urban environment to the cattle sampling area so we considered this population of isolates valid for examining relationships between resistance genes in cattle and human *E. coli* isolates in the study area. The human *E. coli* isolates (*n* = 79), were collected between December 2014 and January 2015 and therefore are concurrent with the sampling frame for the cattle *E. coli* isolates (Mainda et al., 2015). Information on their phenotypes to tested antibiotics is provided as Supplementary Data Sheet S1. Informed consent was obtained from all subjects. All these isolates were submitted for genome sequencing, six did not provide DNA of sufficient quality for AMR gene analysis.

DNA preparation and sequencing methodologies are as published (Mainda et al., 2016). DNA was extracted from the *E. coli* isolates using the Qiagen DNA extraction kit as per manufacturer’s instructions. The DNA was then quantified by Spectrophotometer^®^ Nanodrop and sequenced at Edinburgh Genomics^1^. The Miseq Illumina platform was used for whole genome sequencing which will capture both plasmid and chromosome based sequences with 224 genomes of *E. coli* isolates from cattle and 73 genomes of *E. coli* from humans available for analysis. Quality control of sequence reads was performed using the software FASTQC (Andrews and Fast, 2010) and when necessary the trimming was done with cutadapt (Martin, 2011). The raw reads sequences were *de novo* assembled using SpaDES software (Bankevich et al., 2012).

In addition, for comparative purposes, genome sequences from 317 *E. coli* indicated to have been isolated from humans on the African continent were downloaded from Enterobase (Alikhan et al., 2018) and analyzed as for the Zambian cattle and human isolate sequences in this study. The downloaded sequences were from 15 countries, including Somalia, Egypt, Nigeria, Madagascar, Tanzania, Morocco, Burkina Faso, Senegal, Kenya, Algeria, Mali, Democratic Republic of the Congo, South Africa, Zambia and Gabon. The majority originate from Tanzania relating to studies on *E. coli* across livestock and human samples (*n* = 258) (Moremi et al., 2016; Mshana et al., 2016). The isolates relating to these genomes were collected over a much wider timeframe, from 1973 to 2017. The project numbers for these downloaded genomes are provided as Supplementary Data Sheet S3.

The three sets of genomes in this study were then analyzed for the presence and absence of resistance genes. The resistance genes were initially detected using the Short Read Sequence Type 2 (SRST2) software in a Linux environment (Inouye et al., 2014) but all analyses published here were carried out with outputs obtained for AMR genes within the ResFinder 3.0 database (Zankari et al., 2012). This version of the database included horizontally acquired resistance genes and not resistance conferred by mutations, for example in housekeeping genes. In the current study, acquired AMR genes were analyzed in the context of different *E. coli* phylogroups (Clermont et al., 2015) as *E. coli* has been traditionally clustered by phylogroup and these groupings have a good association with isolates being a commensal or pathogen. The phylotyping method described by Clermont et al. (2013) was performed *in silico*. In short, based on the presence or absence of 4 genes: *chuA*, *yjaA*, *tspE4.C2*, *arpA* isolates initially can be separated into 4 phylogroups (A, B1, B2, D). To further distinguish between groups and assign strains to an additional 4 phylogroups (C, E, F, and cryptic clades) it is necessary to check for the presence of a fifth gene *trpA* and/or distinguish the specific alleles for the above genes. The workflow is as published (Clermont et al., 2013). The identification of the genes and genetic fragments in the isolate genomes was carried out with blastn 2.2.28+ with sequence similarity and length coverage defined as 98 and 99%, respectively. After performing all the steps each *E. coli* sequence was assigned to one of the eight possible phylogroups.

### Statistics

Statistical analyses and visualization were performed in R 3.4.4^2^. *p*-values for resistant genes distribution in different population and in different phylogroups were obtained using “prop.test.” Co-occurrence analysis was carried out using R package “cooccur.” The relationships between different isolates were analyzed using RAxML 8.0.0 with 500 bootstrap samples and the results are presented as phylogenetic trees.

## Results

### Diversity of AMR Genotypes in the Studied *E. coli* Genomes

#### Cattle Isolates

**Figure 1:**
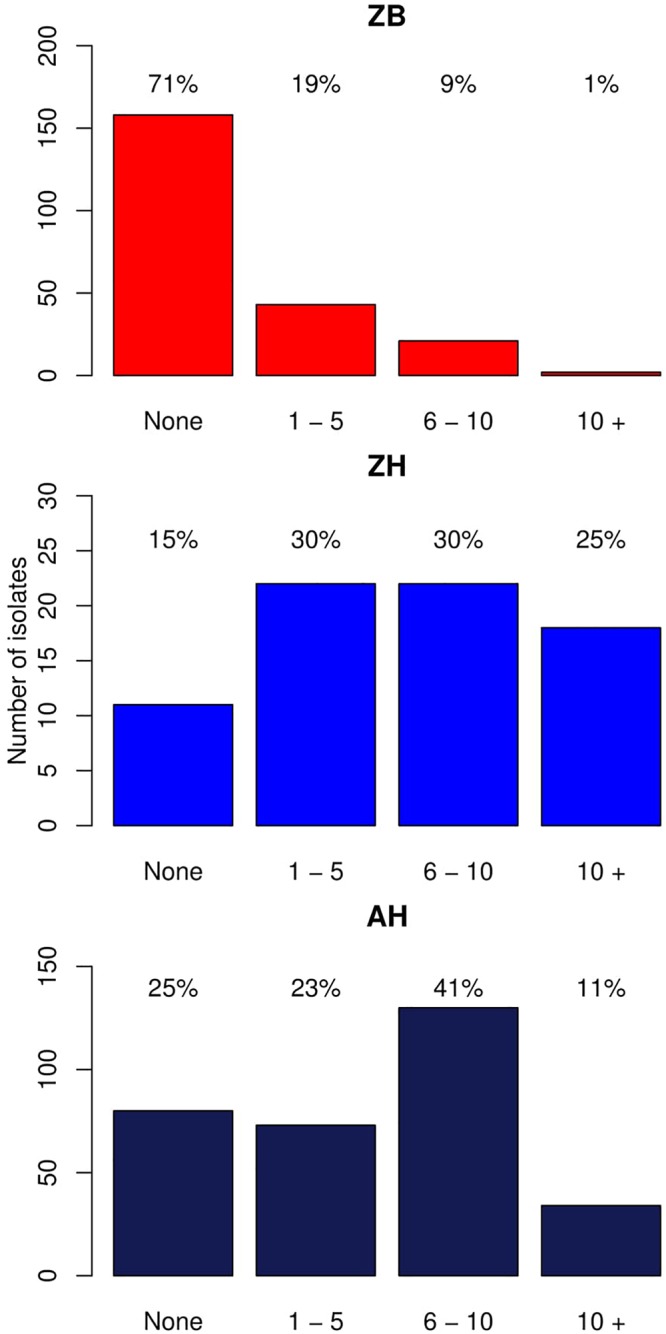
Bar graphs showing the number of isolates with different numbers of acquired resistance genes. ZB is the Zambian bovine cohort; ZH the Zambian human cohort, and AH the isolates from other countries in Africa. The bars show the number of isolates with: 0, 1–5, 6–10, and >10 resistance genes. The percentage of the cohort that these numbers represent are shown above each bar.

As described in the Section “Materials and Methods,” a total of 224 *E. coli* whole genome sequences from cattle were analyzed, which included 61 isolates with established phenotypic resistances to capture resistance gene diversity from our initial pool of 371 cattle isolates (Supplementary Data Sheet S1; Mainda et al., 2015). One phenotypically resistant isolate produced poor quality reads and so was not included. Resistance genes were detected in 66/224 (29.5%) of the genomes, with 51/60 of these from the phenotypically resistant subset. There were 9/60 isolates that were resistant to the antibiotics tested but for which no resistance gene was detected. The six most common resistance genes detected were: *strB1* (19%), *sul2* (17%), *tetA* (16%), *strA4* (13%) *bla*_TEM-1_(13%), and *tetB* (10%). A detailed list of the different resistance genes and their frequencies are presented as Supplementary Data Sheet S4. The numbers and percentages of isolates with 0, 1–5, 6–10, and >10 resistance genes, respectively, are shown Figure 1 (Zambian Bovine–ZB).

#### Human Isolates

Whole genome sequences were analyzed for 73 human isolates from the Lusaka hospital. The phenotypic resistances and comparison where possible with the cattle isolates is presented as Supplementary Data Sheet S1. Where the same antibiotics were tested in both host groups (ampicillin, tetracycline, gentamicin, ciprofloxacin, and cefpodoxime) the human *E. coli* isolates had significantly higher proportions of resistance than the cattle isolates (*p* < 0.0001) for each one (Supplementary Data Sheet S5). Resistance genes were identified in 62/73 (84.9%) of the *E. coli* genomes from humans, although all human isolates had some level of phenotypic resistance to one or more of the antibiotics tested. The frequency and combinations of resistance genes were then analyzed as for the cattle isolates. The six most common resistance genes were: *sul2* (66%), *strB1* (64%), *strA4* (57%), *bla*_TEM-1_ (56%), *aadA* (38%), and *tetA* (32%): gene patterns and frequencies are shown in Supplementary Data Sheet S5.

**Figure 2:**
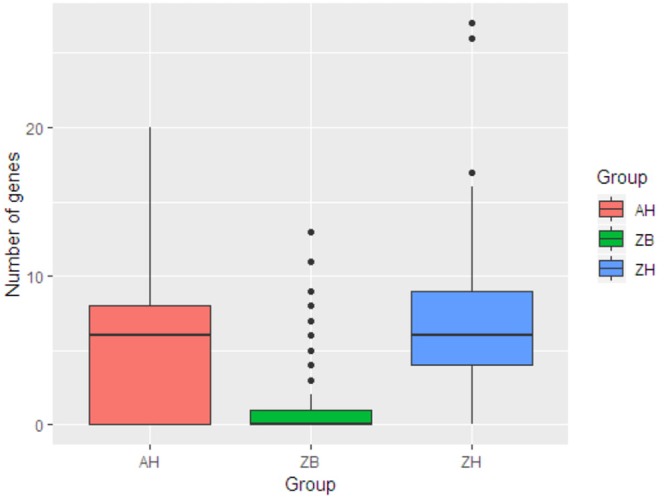
Box plots showing the median values for the number of acquired AMR genes in the three different groups along with the interquartile range. ZB is the Zambian bovine cohort; ZH the Zambian human cohort and AH the isolates from other countries in Africa. The Zambian bovine strains have a significantly reduced number of AMR genes compared to the two human groups (*p* < 0.001).

This was then compared with resistance genes present in 317 human isolates submitted to Enterobase that were from the African continent. From these 237/317 (74.8%) had at least one resistance gene and the patterns plus frequencies are shown in Supplementary Data Sheet S6. The numbers and percentages of isolates with 0, 1–5, 6–10, and >10 resistance genes are plotted in Figure 1 (Zambian Human–ZB and African Human–AH). Despite the sub-sampling bias for WGS toward phenotypically resistant cattle *E. coli* isolates, the carriage of multiple AMR genes was significantly higher in *E. coli* populations from humans (Zambian or African) than in isolates of dairy cattle origin (*p* < 0.001) (Figure 2). The human isolates were therefore much more likely to encode multiple resistance genes.

### Comparison of Acquired AMR Genes From Analyzed Genomes of Cattle and Human *E. coli* Isolates

**Figure 3:**
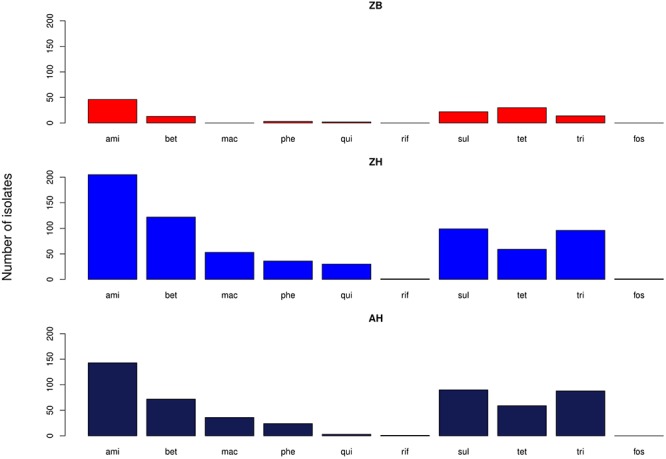
Bar graphs showing the number of isolates in each of the three groups with genes relating to resistance of the specified antibiotic groups. ami – aminoglycosides; bet – Beta-lactams; mac – macrolides; phe – phenolics; qui – quinolones; rif – rifampicin; sul – sulphonamides; tet – tetracyclines; tri – trimethoprim. ZB is the Zambian bovine cohort; ZH the Zambian human cohort and AH the isolates from other countries in Africa.

**Figure 4:**
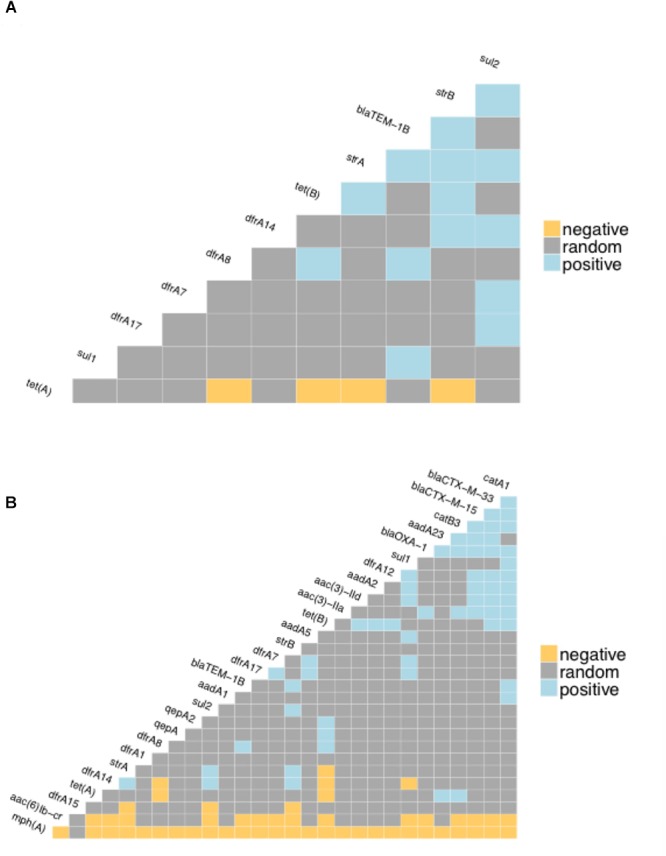
Co-occurrence matrices for acquired antibiotic resistance genes. The matrices show the likelihood of negative or positive co-occurrence of the specified resistance genes among: **(A)** the Zambian bovine *E. coli* genomes; **(B)** the Zambian human genomes. The analysis was carried with the *p* < 0.05 as the threshold for negative or positive.

The proportion of isolates in the different host groups encoding each of the main antibiotic classes was then analyzed (Figure 3). While the general pattern is similar, the overall proportions in the cattle population were lower. Determinants of aminoglycoside resistance were the most frequent gene type identified in either cattle or human *E. coli* populations. Macrolide resistance was relatively absent in the cattle population while, by contrast, tetracycline resistance was over-represented in the cattle isolates (Figure 3). The presence of multiple acquired resistance genes in the same strains and the possibility of particular combinations is important for clinical and molecular epidemiology and to guide antibiotic treatment in a region. In the genomes of *E. coli* isolates from the sampled Zambian dairy cattle, there were 42 different resistance genotype patterns (Supplementary Data Sheet S4) compared with 52 in the Lusaka human isolates (Supplementary Data Sheet S5) and 138 in the wider African human *E. coli* isolates (Supplementary Data Sheet S6). Most of these patterns were not repeated showing the diversity of AMR genotypes captured in this analysis. The most common positive or negative co-associations for resistance genes are shown for the Zambian cattle and Zambian human groups (Figure 4A,B). There were 58 paired co-associations in the Zambian human isolates compared with 14 in the bovine isolates with only 3 being shared between the two groups, these were: *blaTEM-1B* with *strB*; *blaTEM-1B* with *dfrA8*; *strA* with *strB*. This analysis demonstrates the distinct resistance profiles in the *E. coli* strains from the two different hosts and that co-associations are much more likely in human strains that encode multiple resistance genes.

### Phylogenomic Assessment of theResistome of *E. coli* Isolated From Cattleand Humans

**Figure 5:**
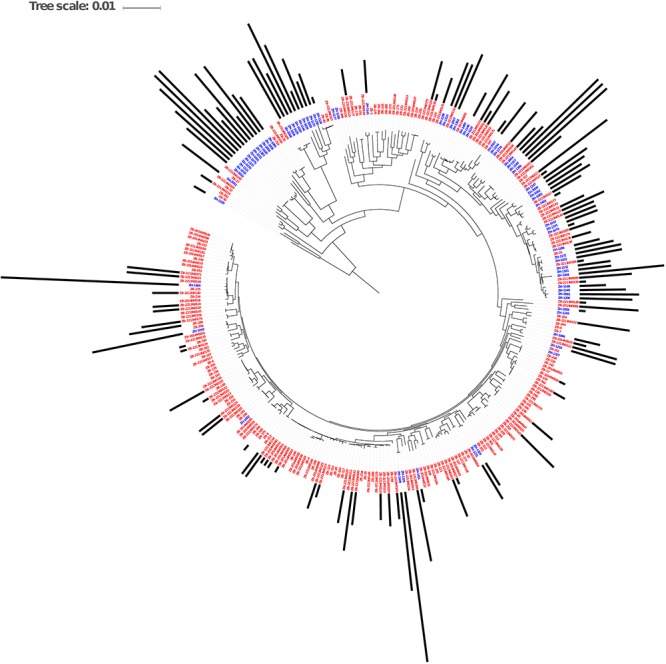
Maximum Likelihood Core SNP based tree of *E. coli* genomes from humans (blue labels, *n* = 77) and cattle (red labels, *n* = 186) showing resistance gene carriage. The black bars show the number of acquired AMR genes in each genome. The bars are based on the scale range of AMR genes between 0 and 17 per genome.

**Figure 6:**
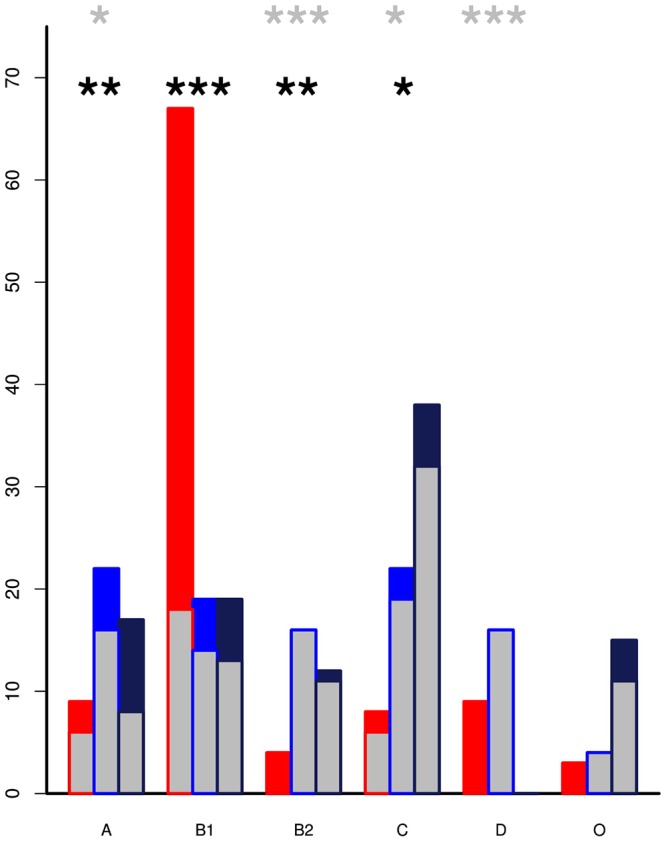
The percentage of isolates by phylogroup with inset showing the proportion that encode acquired AMR genes. The Zambian cattle isolates (red) are predominately in phylogroup B1 but are associated with significantly lower levels of AMR genes than the Zambian human (blue) and All Africa human (black) groups. Pathogenic strains are more likely to be clustered in the B2 and D phylogroups. Within these there were relatively low percentages of cattle isolates but these had detectable AMR genes by comparison to high resistance levels in the human clusters. *p*-values from pairwise *t*-tests: the black asterisks mark significance for proportion of strains in each phylogroup while the gray asterisks mark significance for the level of resistance in each phylogroup ^∗^ < 0.01, ^∗∗^ < 0.001, ^∗∗∗^ < 0.0001. Only statistics between ZB and ZH are shown.

The genomes from the two Zambian groups were clustered according to single nucleotide polymorphisms in core genes (Figure 5). The genomes from the Zambian human *E. coli* isolates tended to cluster together often away from those of cattle origin and this in part reflects differences in phylogroups between the two Zambian sample sets (Figure 6). The distribution and number of acquired resistance genes were then plotted on to the relationship tree and this provides a clear visual representation of the multiple resistance genes in the Zambian human compared to the bovine *E. coli* isolates (Figure 5). The majority of cattle *E. coli* with resistance genes were assigned to phylogroup B1 indicating that these isolates were largely commensals (Figure 6). However, the isolates with multiple AMR resistance genes from the Zambian human isolates were distributed across the major phylogroups including those associated with more pathogenic strains (Phylogroups B2 and D). The human strains originating from across Africa followed a similar trend to the group of Zambian human isolates although no phylogroup D strains were detected. It is striking that the bovine *E. coli* isolates in phylogroups associated with pathogenesis, B2 and D, had negligible AMR levels in contrast to the human isolates in these groups (Figure 6). Conversely, the proportion of isolates with AMR genes in the “commensal” B1 cluster was significantly higher for the two sets of sequenced human isolates compared to the sequenced bovine isolates.

## Discussion

Our previous study examined antibiotic use in cattle on different sized dairy units, including smallholdings in a region surrounding Lusaka in Zambia. That study demonstrated phenotypic resistance commensurate with tetracycline and penicillins as the main antibiotics used in cattle in this region, with the majority of isolates exhibiting no resistance to the antibiotics tested (Mainda et al., 2015). All resistant isolates and randomly selected non-resistant isolates from our previous study were sequenced here to obtain genomic information in relation to the acquired resistance genes present. During the study we were also able to obtain a group of human *E. coli* isolates selected from people presenting with diarrhea at a University Teaching Hospital in Lusaka over the same timeframe (2014). Phenotypic testing for resistance demonstrated significantly higher levels of resistance amongst the human isolates when the same antibiotics were tested (Supplementary Data Sheet S1). Sequencing of these isolates then provided an opportunity to compare the type and distribution of acquired resistance genes in *E. coli* isolates originating from the two hosts. While we appreciate the caveats of such an analysis, especially the relatively small number of human isolates and their *post hoc* addition, we consider the comparative analysis has real value to address questions around resistance gene epidemiology in *E. coli* as a sentinel species in the two hosts from this area of Africa. To bolster the analysis, we have also included comparison with 317 *E. coli* genomes isolated from humans in different African countries. In general, these have provided similar results to the Zambian human cohort which we consider as a validation of this study.

While we would not anticipate *E. coli* to be the aetiological agent responsible for diarrhea in the majority of patients, it would be reasonable to expect that the human cohort may contain a higher proportion of potentially pathogenic *E. coli* compared to the cattle set. This was apparent from an analysis of the phylogroups of the isolates (Figure 6) with, in fact, both human cohorts containing higher proportions of phylogroups associated with pathogenic strains (B2 and/or D) when compared with the cattle isolates which were predominately phylogroup B1, more associated with a commensal existence. While we do not have the antimicrobial treatment data for the Zambian human isolates we would anticipate that we are still capturing many isolates in these groups that are not associated with disease.

Despite the pre-selection of all phenotypically resistant isolates for sequencing in the cattle set it was evident that resistance genes were significantly more frequent in the *E. coli* isolates from the Zambian human population compared to *E. coli* isolates from local dairy cattle. The majority of human isolates encoded resistance genes in stark contrast to the cattle isolates and the human isolates carried a significantly higher mean number of resistance genes than the cattle isolates (Figure 1). As a consequence, *E. coli* isolates encoding multiple drug resistance were much more common in the human cohort. The human isolates also had a significantly higher frequency of acquired AMR genes compared to the cattle isolates even when considering those assigned only to phylogroup B1, generally associated more with commensal strains.

There has been considerable debate over the emergence of AMR in humans that may be linked to antibiotic use in production animals (Goodyear, 2002; Gillings, 2013; O’Neill, 2014; Woolhouse et al., 2015; van Bunnik and Woolhouse, 2017). Whilst this study covers only one area of one country, it is evident from our findings that the resistance genotypes present in the human isolates were much more diverse than those found in cattle with resistance genes identified in the human isolates not found in cattle isolates. In general there was no clear evidence to indicate exchange of combined AMR genes between the two hosts or sharing of specific resistant strains. However, many AMR genes are present on highly mobilizable genetic elements including plasmids (Woolhouse et al., 2015) so ready exchange of these to generate different AMR combinations in different strains can occur and the original sources of such genes is virtually impossible to define. Beyond this and a study in poultry in Zambia (Chishimba et al., 2016) the significance of other reservoirs of AMR in the region have not been investigated. In addition, while we have collected data on the types of antibiotics used in the cattle population (Mainda et al., 2015), we do not have equivalent data for other production systems and the human population in Lusaka and so the selection pressures in these communities on AMR by antibiotic use are not defined (Mshana et al., 2013).

Despite this lack of information our study indicates that the *E. coli* of human origin and those of cattle origins are under different and largely independent AMR selection pressures. This is evidenced by the presence of rare but important clinical AMR genes encoding beta lactamases such as *bla*_NDM_, *bla*_CTX-M_, *bla*_OXA_ and fluoroquinolones resistance encoding genes *qnr*, *aac* in *E. coli* of human origin that were absent in the *E. coli* of cattle origin. However, the resistance genes encoding older and commonly applied antibiotics such as *tetA* and *tetB* for tetracycline, *strA* and *strB* for streptomycin, *sul1* and *sul2* for sulphonamides and *dfrA* for trimethoprim were detected in both populations, which is an indication that such resistances are common. However, such selection could still be driven by independent pressures in each population rather than cross-over between them. It is also useful evidence that certain clinically important antibiotics such as cephalosporins and fluoroquinolones are still being mostly used in the treatment of human infection and rarely used in dairy cattle of the study area. This could be attributed to the high cost of such antibiotics when compared to the first generation antibiotics (tetracycline, penicillin, and sulphonamides) which are less expensive and the ones commonly used in cattle (Mainda et al., 2015). Further, the human hosts were likely to have been treated with different types of antibiotics than the cattle host as shown by the wide diversity of resistance genes.

A recent study that examined antibiotic resistance in atypical enteropathogenic *Escherichia coli* from both sub-Saharan Africa and Asia (Ingle et al., 2018) indicated high levels of AMR in these human isolates concordant with our study. In particular they noted certain co-associations, particularly of *strA* and *strB* with *sul2*, with *blaTEM* and *dfrA14* being linked in a further subset. These associations match those shown to be prevalent in our study in both cattle and human isolates and probably indicate the longer term evolution of combined resistances to antibiotics that have been applied over longer timeframes.

More studies need to be carried out to analyze the distribution of strains and resistance genes in species that are in physical co-association as even in the context of *E. coli* we are still trying to determine if AMR transfer is primarily one of strain acquisition and strain maintenance in that host or, perhaps more likely, temporary transmission to a new host and then dissemination of the AMR gene(s) to a more host-adapted strain. This dynamic will vary depending on the bacterial species being investigated, but species that we know can be promiscuous and zoonotic, such as *E. coli*, are a logical point of study until metagenome sequencing costs allow a wider picture of AMR dynamics in complex populations to be gathered. Even then, better methods need to be developed to associate AMR genes with the carrying organism.

The limited sharing of strains and resistance genes between the dairy cattle and human populations in the study area and sampling interval serves as an important reminder of the challenge of establishing transmission routes, and of attributing the AMR problem in humans to antibiotics use in farmed animals. As genome sequence data accrue it should be feasible to understand the direction, nature and frequency of gene flow between animal and human populations in greater detail. At the same time, metadata related to antibiotic use will be key to understand the selective pressures and impact of management strategies. Our main message from this study is that some agricultural sectors are behaving responsibly through both choice and necessity which helps maintain the incredible value of many antimicrobials for treatment of livestock diseases which reduce disease, improve animal welfare and help maintain the livelihoods of the many smallholders and farmers which are dependent on their productivity.

## Ethics Statement

This study was carried out in accordance with the recommendations of the, Tropical Diseases Research Centre (TDRC) Ethics Review Committee guidelines at https://healthresearchweb.org/en/zambia/ethics_1007, with written informed consent from all subjects. All subjects gave written informed consent in accordance with the Declaration of Helsinki. The study involving human isolates was approved by the “Tropical Diseases Research Centre Ethical Committee” Clearance #: STC/2015/12. The animal sampling study was carried out in accordance with the recommendations of the University of Edinburgh’s “Animal Welfare and Ethics Committee” (AWERB) at https://www.ed.ac.uk/research/animal-research/animal-welfare-ethics and the protocol was approved by the UoE AWERB. The project work also aligned to permissions under a licence to Professor Mark Stevens: Mark Stevens PPL 60/4420.

## Author Contributions

GM, LS, PB, MS, BB, JM, and DG conceived and planned the experiments. GM, LS, DG, SM, and GK carried out the field work and microbiology. NL, GM, ER, and PB carried out the main sequence and data analyses. GM, NL, and DG contributed to the interpretation of the results. GM and DG took the lead in writing the manuscript with most figures produced by NL. All authors provided critical feedback and helped shape the research, analysis, and manuscript.

## Conflict of Interest Statement

The authors declare that the research was conducted in the absence of any commercial or financial relationships that could be construed as a potential conflict of interest.

## Footnotes

1 http://genomics.ed.ac.uk/

2 www.r-project.org/
