# Supplementary material for: Whole Genome Sequence Analysis Reveals Lower Diversity and Frequency of Acquired Antimicrobial Resistance (AMR) Genes in E. coli From Dairy Herds Compared With Human Isolates From the Same Region of Central Zambia
Source: Front Microbiol. 2019 May 31;10:1114. doi: 10.3389/fmicb.2019.01114 (PMC6555227; doi:10.3389/fmicb.2019.01114)
Supplement: Supplementary file 2 [file Data_Sheet_2.pdf]

Supplementary Data S2

Selection of *E. coli* isolates for genome sequencing

Zambian cattle *E. coli* isolates (ZB)

371 cattle *E. coli i*solates from 110 farms, each isolate from separate animals (8)

All isolates tested for phenotypic resistance to 6 different antibiotics (8) And Supp. Data S1

61 resistant to at least one tested antibiotic; all these were sequenced

310 were phenotypically sensitive

166 of these sensitive isolates were sequenced. 125 selected at random plus 41 PCR +ve for *stx* in another study (10)

In total **227** cattle *E. coli* isolates were sequenced from the 371. **224** produced data of sufficient quality for analysis

Zambian human *E. coli* isolates (ZH)

79 human *E. coli i*solates from different patients

All isolates tested for phenotypic resistance to 13 different antibiotics (Supp. Data S1)

All resistant to at least one tested antibiotic; all isolates were sequenced

73 produced data of sufficient quality for analysis
